# Structural modification of the *Pseudomonas aeruginosa* alkylquinoline cell–cell communication signal, HHQ, leads to benzofuranoquinolines with anti-virulence behaviour in ESKAPE pathogens

**DOI:** 10.1099/mic.0.001303

**Published:** 2023-03-02

**Authors:** Veronica Rossetto, Ay'sha Moore-Machacek, David F. Woods, Helena M. Galvão, Rachel M. Shanahan, Aobha Hickey, Niall O’Leary, Fergal O’Gara, Gerard P. McGlacken, F. Jerry Reen

**Affiliations:** ^1^​ Faculty of Science and Technology, Universidade do Algarve, Algarve, Portugal; ^2^​ School of Microbiology, University College Cork, Cork, Ireland; ^3^​ School of Chemistry and Analytical and Biological Chemistry Research Facility, University College Cork, Cork, Ireland; ^4^​ Biomerit Research Centre, School of Microbiology, University College Cork, Cork, Ireland; ^5^​ Wal-yan Respiratory Research Centre, Telethon Kids Institute, Perth, WA, Australia; ^6^​ Synthesis and Solid State Pharmaceutical Centre, University College Cork, Cork, Ireland

**Keywords:** antimicrobial resistance, anti-virulence, biofilm, ESKAPE pathogens, HHQ, *Pseudomonas aeruginosa*

## Abstract

Microbial populations have evolved intricate networks of negotiation and communication through which they can coexist in natural and host ecosystems. The nature of these systems can be complex and they are, for the most part, poorly understood at the polymicrobial level. The Pseudomonas Quinolone Signal (PQS) and its precursor 4-hydroxy-2-heptylquinoline (HHQ) are signal molecules produced by the important nosocomial pathogen *Pseudomonas aeruginosa *. They are known to modulate the behaviour of co-colonizing bacterial and fungal pathogens such as *Bacillus atropheaus*, *Candida albicans* and *Aspergillus fumigatus*. While the structural basis for alkyl-quinolone signalling within *P. aeruginosa* has been studied extensively, less is known about how structural derivatives of these molecules can influence multicellular behaviour and population-level decision-making in other co-colonizing organisms. In this study, we investigated a suite of small molecules derived initially from the HHQ framework, for anti-virulence activity against ESKAPE pathogens, at the species and strain levels. Somewhat surprisingly, with appropriate substitution, loss of the alkyl chain (present in HHQ and PQS) did not result in a loss of activity, presenting a more easily accessible synthetic framework for investigation. Virulence profiling uncovered significant levels of inter-strain variation among the responses of clinical and environmental isolates to small-molecule challenge. While several lead compounds were identified in this study, further work is needed to appreciate the extent of strain-level tolerance to small-molecule anti-infectives among pathogenic organisms.

## Introduction

The association between microbial life and eukaryotic organisms has evolved to a state where coexistence and even co-dependence on the part of multicellular organisms has become the natural order. Humans, animals, plants, insects and other organisms typically depend on complex microbial communities for the provision of key traits and factors that sustain health, growth and propagation. In many cases, these relationships have evolved to various forms of symbiosis, from commensalism in the human gut [1] to mutualism in the form of the *Allivibrio fischeri* – *Euprymna scolopes* interdependence [2, 3]. However, mutualism and parasitism are often viewed as two ends of the same paradigm, and a shift in dynamics caused by changes in the external environment can lead to situations where the presence of keystone microbial populations can become detrimental to the host [4]. It is often within these situations that opportunistic pathogens such as the ESKAPE pathogens emerge, causing morbidity and mortality in patients with underlying conditions. The ESKAPE acronym refers to six nosocomial pathogens in which resistance to conventional antibiotics is particularly prevalent and increasing: *Enterococcus faecium *, *Staphylococcus aureus *, *Klebsiella pneumoniae *, *Acinetobacter baumanii*, *Pseudomonas aeruginosa* and *Enterobacter* sp. Various strategies to control infection by ESKAPE pathogens have been pursued apart from antibiotics, including but not limited to bacteriophages [5], medicinal plant extracts [6], non-thermal plasma treatment [7], adjuvants [8], violet and blue light [9], and nanoparticles [10].

The virulence systems of ESKAPE pathogens have been extensively studied, yet the complex decision-making processes in these microbes that govern the balance between benign behaviour and the virulent state remain less well understood. Hierarchical networks of transcriptional and post-transcriptional proteins work in tandem with small RNAs and second messengers to ensure cells can respond to changes in their external environment during colonization. Key to this is the ability to make decisions at the population level, a phenomenon known as quorum sensing. First described through the lens of auto-induction, the current understanding of how cells communicate and co-ordinate behaviours at the population level has advanced significantly from its initial inception [11–14]. In particular, the understanding that quorum sensing was ‘more than just a numbers game’ [15] led to new theories and descriptors for just how microbes engage each other in the form of chemical communication [16]. Expansion of the quorum sensing realm to include interspecies signalling added further complexity to the already intricate ligand–protein interactions, notwithstanding the importance of differentiating signals from cues and coercive behaviour [17]. Interspecies signalling occurs in all orders of life [18], and the extent to which microbes can both govern and respond to control from other species is the subject of much investigation [19–21]. It stands to reason that the evolution of interspecies communication would arrive at signalling outputs produced by a minority (ranging from, for example, spoken languages to chemical messages), which could be detected or decoded by many, a behavioural phenomenon seen in humans, animals, plants and other organisms. Thus, chemical communication presents an opportunity for control of microbial behaviour beyond the classical growth-limiting effects of conventional antibiotics [16, 22]. The alkyl-quinolone signalling system in *P. aeruginosa* is one example of an interspecies molecule that can control multicellular behaviour in a broad spectrum of fungal and bacterial pathogens [23, 24]. Several reports have described its potential as a virulence control mechanism in microbial systems, whereby the formation of aggregate pathogen communities called biofilms is particularly affected by the quorum sensing process [25–30].

Biofilm formation in bacterial and fungal pathogens has been described as a ‘breeding ground’ for the emergence and spread of antimicrobial resistance. Biofilms promote tolerance to conventional and host-derived antibiotic agents, enable cell–cell transfer of resistance genes, and facilitate persistence and chronic colonization in susceptible hosts [31]. Resulting from a multi-stage process initiated upon adhesion to a biotic or abiotic surface, or indeed to other cells, biofilms encased in an extracellular matrix have been shown to be industrially [32] and clinically [33, 34] relevant, and they remain a serious public health concern. The molecular mechanism underpinning biofilm formation in microbial systems is complex and varied, is dependent on environmental conditions, and is typically polymicrobial [35, 36]. Recent studies at single-cell resolution suggest division of labour within these complex aggregates, reporting that physiological states can coexist micrometres apart within the same biofilm [37]. The extent to which spatial heterogeneity exists within these complex aggregates, and the role of factors such as the extracellular matrix, species composition and nutrient availability in shaping these organized structures remains to be established [38]. What is clear is that biofilms represent a significant challenge to the clinical management of disease, and sustainable strategies to counteract the formation of these tolerant structures are urgently required [31, 39].

A potential ‘Achilles Heel’ in the formation of biofilms is the importance of cell to cell communication at the intra-species, inter-species, and inter-kingdom dimension, for example within mixed fungal–bacterial biofilms [40]. Co-ordinating cellular behaviour and decision-making at the population level in biofilms requires a complex hierarchy of signal transduction systems to switch on and off the required genes in response to spatial, temporal and external cues. Therefore, strategies that seek to disable co-ordination of these behaviours could potentially deprive microbial communities of this key defence strategy. While this strategy is not new, the use of signal molecules from dominant nosocomial pathogens as the framework for control of competing pathogens is an innovative approach. Using a language that has evolved over time in successful pathogens could offer an alternative approach for pathogen control, notwithstanding the significant challenges that remain in tackling polymicrobial communities and in engineering a microbial signal molecule to be druggable [41].

**Fig. 1.**
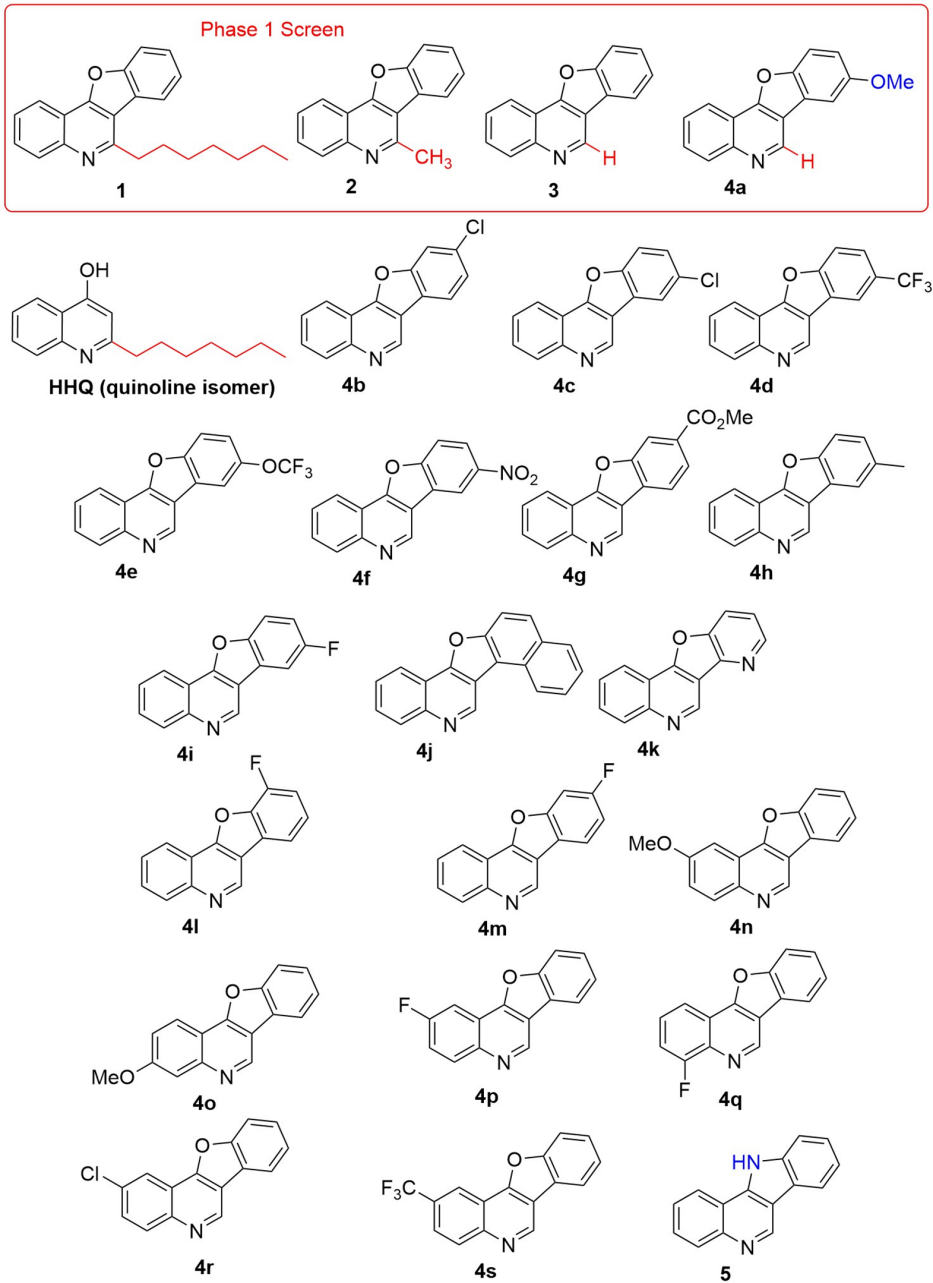
Substrate scope of benzofuroquinoline structures used in this study.

We started with a 4-hydroxy-2-heptylquinoline (HHQ)-based framework devoid of a 4-OH group, but with the O tied up as an ether (compound **1**, Fig. 1). We found that the heptyl chain present in HHQ was not always required for activity in our tests. Thus, we pursued more easily accessible derivatives, with smaller (CH_3_) or no (H) alkyl chain at C2, with the biological potential for control of species-specific behaviours in microbial pathogens [42]. In addition to the ESKAPE pathogens, several non-*aureus Staphylococcus* species were chosen to investigate the broad activity of the compounds, while *Bacillus atropheaus* was included on the basis that it extends the ecological relevance of these compounds to a soil organism previously reported to be influenced by the Pseudomonas Quinolone Signal (PQS) and its biological precursor HHQ [23, 27, 30]. Several of the compounds possess significant anti-biofilm, anti-virulence and in some cases growth-inhibitory activities against key clinical pathogens and thus warrant further investigation. An emerging theme of heterogeneous strain responses to anti-biofilm formation activity of the molecules was observed, which raises key questions relating to the development and implementation of anti-virulence strategies for infection control.

## Methods

### Bacterial strains, culture conditions and chemical compounds

A list of bacterial strains included in this study is provided in Table 1. All species were grown in/on tryptic soy broth (TSB) or agar (TSA) as required for routine maintenance and phenotypic assay. All strains were grown and tested at 37 °C with the exception of *B. atropheaus* which was grown and tested at 30 °C.

**Table 1.** Strains included in this study

Species	Strain	Reference
* E. faecalis *	C8	Brine, UCC*
* S. aureus *	NCDO 949 MorphT MorphD VISA MRSA	Culture Collection Clinical, UCC Clinical, UCC Clinical, UCC Clinical, UCC
* S. haemolyticus *	CUH-T LBMJ M-	Clinical, UCC Clinical, UCC Clinical, UCC
* S. epidermidis *	B1.9 MorphK MorphD	Environmental, UCC Clinical, UCC Clinical, UCC
* S. equorum *	B1.2 B1.2’ B1.6	Environmental, UCC Environmental, UCC Environmental, UCC
* S. hominis *	B1.29	Environmental, UCC
* P. aeruginosa *	PA14	[70]
* K. pneumoniae *	28 296	[71]
*A. baumanii*	#109 #446	Clinical, UCC Clinical, UCC
*B. atropheaus*	NCTC 10073	Culture Collection

*UCC, University College Cork.

Synthesis of the compounds used in this study has previously been described in Shanahan *et al*. [42] and involved a modified Pd-catalysed intramolecular CH functionalization reaction (https://doi.org/10.1016/j.tet.2015.12.039) and the key cyclization step. All compounds were solubilized in DMSO and stored at 4 °C until use. Unless otherwise stated, all compounds were tested at a concentration of 30 µM (Fig. 1).

### Biofilm assay

Overnight cultures adjusted to an OD_600nm_ of 0.05 in (200 µl) TSB were supplemented with each compound at 30 µM or an equal volume of the carrier control (DMSO) and incubated in microtitre plates at 37 °C (* Staphylococcus* spp., *P. aeruginosa *, *K. pneumoniae *, *E. faecalis* and *A. baumanii*) or 30 °C (*B. atropheaus*) for 18 h. Following incubation, plates were removed to room temperature and washed in distilled water to removed non-adhered cells, after which crystal violet (0.1 %, w/v) was added to each well for 30 min. Following repeated washing, the attached crystal violet was solubilized with ethanol and quantified spectrophotometrically at Abs_595nm_. Controls included in these assays were TSB medium without inoculum and DMSO as a carrier control. Assays were also performed as above in 24-well plates with 1 ml volumes used per well. Experiments were performed with multiple technical replicates and with at least three independent biological replicates.

### Bacterial growth curves

Growth curve assays were designed to mimic the set-up of the biofilm assays. Briefly, overnight TSB cultures were adjusted to an OD_600nm_ of 0.05 in fresh TSB medium, in the presence and absence of 30 µM of each compound, with DMSO as a carrier control. Growth was measured spectrophotometrically at OD_600nm_ in honeycomb plates (performed in triplicate and with three independent biological replicates for each strain) incubated at 37 °C for *Staphylococcus* spp. and *P. aeruginosa* PA14, and at 30 °C for *B. atrophaeus *, using a Bioscreen-C automated growth curve analysis system to capture the biomass data. The honeycomb plates (Growth Curves USA) are designed for the Bioscreen-C platform, consisting of 10×10 wells, with a maximum volume of 400 µl.

### Swarming motility assay

Motility was measured on TSA plates with 0.3 % agar (w/v) for *B. atropheaus* and on Eiken Agar (Eiken Chemical Tokyo) 0.8 % (w/v) for *P. aeruginosa* PA14, in the presence and absence of test compounds. Briefly, sterile tips were used to gently inoculate a single colony onto the surface of media plates with minimal pressure. Motility plates supplemented with DMSO and untreated plates were included as controls. Plates were incubated overnight (37 °C for *P. aeruginosa* PA14 or 30 °C for *B. atrophaeus *) and motility was observed as the distance of the swarm front following overnight incubation.

### Pyocyanin assay

Pyocyanin was extracted as described previously [43]. Overnight cultures of *P. aeruginosa* PA14 were adjusted to an OD_600nm_ of 0.05 in TSB, supplemented with compounds (30 µM), and incubated at 37 °C with shaking at 180 rpm. Samples (5 ml) were removed and underwent centrifugation at 5000 rpm for 15 min to separate cells from the supernatant. Chloroform (3 ml) was added, and the tubes were vortexed for 30 s and centrifuged at 5000 rpm for 5 min. The organic phase was transferred to a fresh tube using a pipette and 2 ml of 0.2 M HCl was added. Tubes were vortexed and, following centrifugation at 5000 rpm for 5 min, the absorbance of the top pink phase was read at OD_520nm_ and the concentration was corrected according to the formula: (Abs_520nm_×17.072)×1.5 (dilution factor of the acidic phase).

### Statistical analyses

Data presentation was performed using GraphPad Prism 8. The results were statistically analysed with one-way ANOVA followed by Dunnett’s multiple comparison test (GraphPad Prism 8). Differences between tested compounds were considered significant at a significance level of *P*<0.05.

## Results

### HHQ derivatives suppress key virulence phenotypes in *P. aeruginosa*

Among the three types of known quorum sensing mechanisms in *P. aeruginosa *, the PQS system has emerged as a modulator of interspecies and inter-kingdom behaviour in bacteria and fungi [27–29]. It follows that derivatives of these signal molecules may prove to be effective modulators of pathogen behaviour, both in *P. aeruginosa* and in competing organisms. The benzofuroquinoline framework containing a seven-carbon side chain was initially chosen, as it maps onto HHQ quite well, yet is devoid of the free −OH group (Fig. 1). Thus, compound **1** was initially evaluated for its ability to influence biofilm formation and swarming motility in *P. aeruginosa* PA14. Growth, initiated from planktonic cultures to mimic the conditions of the biofilm analysis, was also measured to ensure a phenotypic response was not simply a result of limiting biomass.

**Fig. 2.**
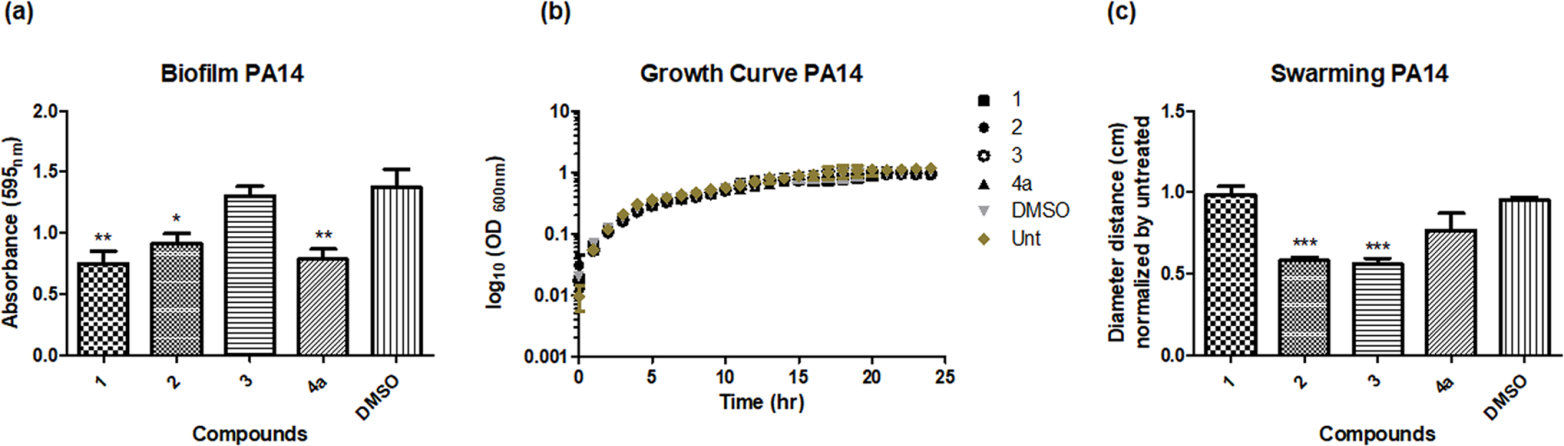
Activity analysis of benzofuroquinoline compounds (30 µM) against *P. aeruginosa* PA14. (**a**) Biofilm assay presented as crystal violet biomass Abs_595nm_. (**b**) Growth curve analysis in the presence of compounds and DMSO carrier control. (**c**) Swarming motility on Eiken agar plates measured as the swarm distance from the tips of the outermost tendrils characteristic of this strain and normalized to the untreated control. In all cases, data represent the mean (±sem) of at least three independent biological replicates. Statistical significance (one-way ANOVA with Dunnett’s Multiple Comparison test) is presented relative to the DMSO carrier control (*P≤0.05, **P≤0.005, ***P≤0.001).

Addition of compound **1** resulted in significantly reduced biofilm production compared to the DMSO control, but no effect on swarming activity was observed (Fig. 2). While both activities require multicellular cooperation, they are governed by distinct regulatory systems. To investigate the structural features responsible for the activity, and specifically the role of the alkyl chain, three additional molecules were tested: compound **2** with a methyl group in place of the alkyl side chain, compound **3** with a H in position of the alkyl side chain, and compound **4a** with a H in position of the alkyl side chain and methoxy group substitution. Replacement of the alkyl chain with a H (in **2** and **3**) led to loss of anti-biofilm activity, but surprisingly, anti-biofilm activity was restored upon methoxy substitution in **4a**. Similarly, shortening (**2**) or loss (**3**) of the alkyl chain led to anti-swarming activity against *P. aeruginosa *, which was lost upon methoxy substitution in **4a** (Fig. 2). These distinct phenotypic influences, which were found to be independent of growth inhibition (Fig. 2), suggested that the molecular mechanism underpinning the observed phenotypes may occur through distinct molecular systems. The initial results also presented us with a more synthetically accessible suite of compounds whereby the alkyl chain was not a requirement for anti-biofilm activity. Synthesis of these types of compounds possessing long alkyl chains tend to be more difficult to prepare and characterize, and ultimately they are less druggable.

**Fig. 3.**
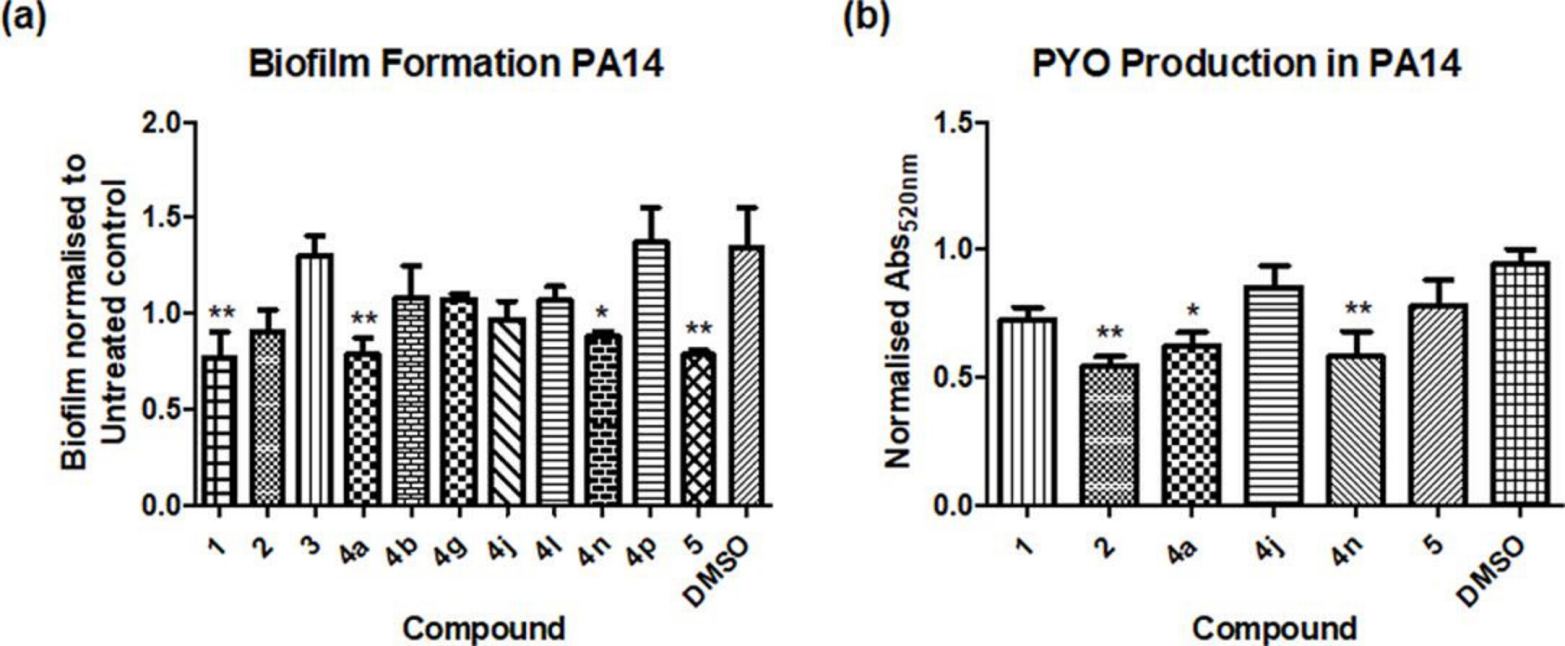
(**a**) Biofilm assay normalized to untreated control. (**b**) Pyocyanin extraction normalized to the untreated control. Data presented are the mean (±sem) of at least three independent biological replicates. Significant differences were determined by one-way ANOVA with Dunnett’s multiple comparison test (*P≤0.05, **P≤0.005).

Thus, the strong anti-biofilm activity of **4a** led us to synthetize a suite of synthetic derivatives of the compound **3** framework in which the alkyl chain had been replaced by a H group in addition to further substitution of the ring structure. Biofilm formation in *P. aeruginosa* PA14 was inhibited by 30 µM concentrations of derivative compounds **1**, **4a**, **4n** and **5** (Figs 3a and S1, available in the online version of this article).

While biofilm formation is associated with the chronic lifestyle of infection in *P. aeruginosa *, pyocyanin (PYO) production is linked to acute systemic infection and plays a significant role in the pathophysiology of *P. aeruginosa* infections. Each of the compounds was tested for its ability to influence PYO production in aerobically grown *P. aeruginosa,* and compounds **2**, **4a** and **4n** were shown to significantly reduce PYO production relative to the DMSO control (Fig. 3b). It was interesting to note that compounds **1** and **5**, which were effective in suppressing biofilm in PA14, had no impact on PYO production, suggesting an independent mechanism of control.

### Species-specific modulation of biofilm formation in ESKAPE pathogens

Communication within species has been shown to be integral to coordinating collective behaviour. However, it is known that this perception of signalling molecules occurs at the interspecies level as well. Therefore, we studied the impact of these compounds on three human and soil pathogenic species: *S. aureus* NCDO949, *S. haemolyticus* CUH-T and *B. atropheaus* NCTC10073 that are known to coexist in natural and clinical niches with *P. aeruginosa *. Encouragingly, the lead compounds identified in the *P. aeruginosa* assays were again found to exhibit species-specific anti-biofilm activity in these assays.

**Fig. 4.**
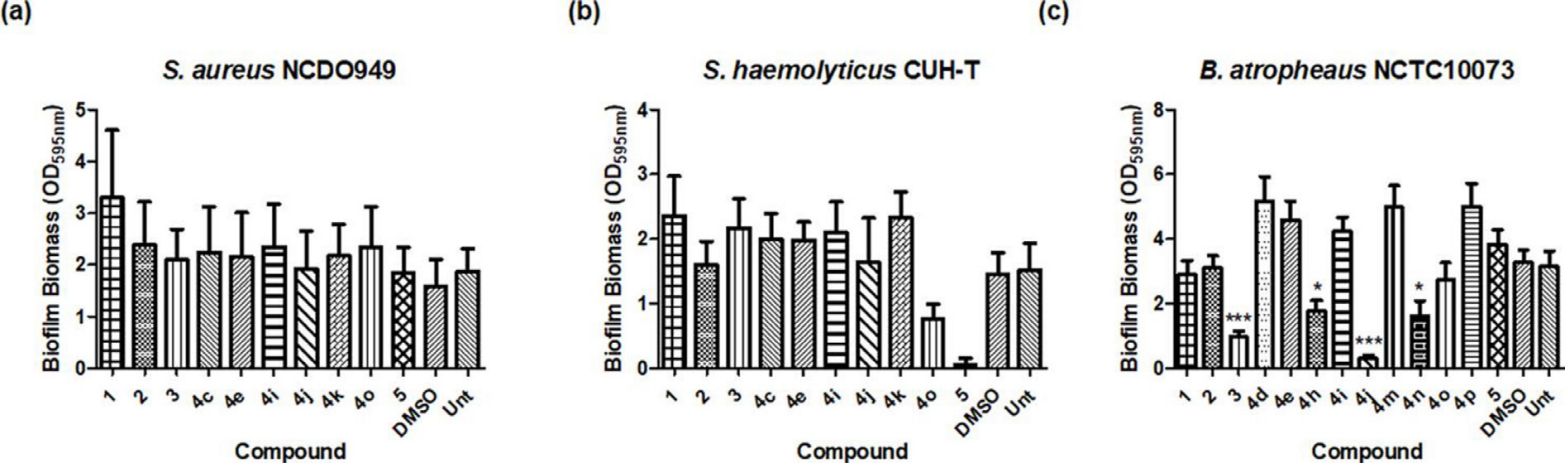
Biofilm formation in (**a**) *S. aureus *, (**b**) *S. haemolyticus* and (**c**) *B. atropheaus* strains as measured in 96-well plates with crystal violet staining. All data presented are the mean (±sem) of at least five independent biological replicates. Statistical analysis was performed by one-way ANOVA with Dunnett’s Multiple Comparison test (*P≤0.05, ***P≤0.001). In each panel, Unt refers to the untreated control.

While none of the compounds tested influenced biofilm formation in the *S. aureus* NCDO949 strain, compounds **4o** and **5** had a significant impact (Student’s *t*-test, *P*=0.0077 and *P*=0.0098, respectively) on the formation of biofilms by the *S. haemolyticus* strain (Figs 4a, b and S2). Biofilm formation was completely abolished in the *S. haemolyticus* strain in the presence of compound **5**, being among the most potent activities identified in this study. In the case of *B. atropheaus*, compound **4j** exhibited the most potent anti-biofilm activity, followed by **4n**, **4h** and **3**, further indicating that distinct mechanisms are involved at the species level with respect to these compound–phenotype correlations (Figs 4c and S2).

**Fig. 5.**
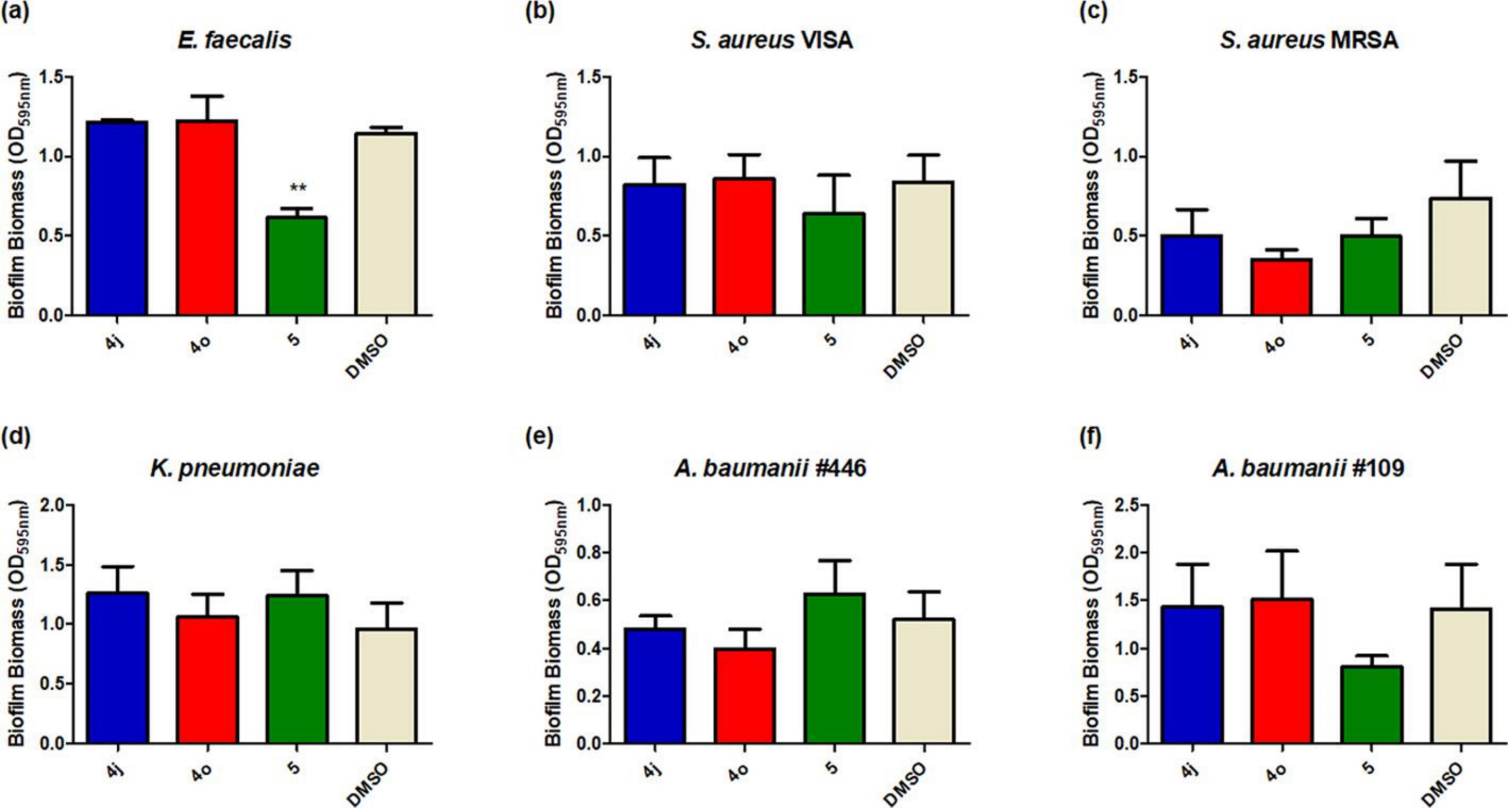
Biofilm formation in *E. faecalis* and ESKAPE pathogens in the presence of lead compounds (30 µM) **4j**, 4o and 5. All data presented are the mean (±sem) of at least three independent biological replicates. Statistical analysis was performed by one-way ANOVA with Dunnett’s Multiple Comparison testing (**P≤0.005).

Following on with three lead compounds (**4j**, **4o** and **5**), we studied biofilm formation in *E. faecalis* and the remaining ESKAPE pathogens *K. pneumoniae* and *A. baumanii* (Fig. 5). Biofilm formation in *E. faecalis* was reduced in the presence of compound **5** (*P*<0.05) while **4o** was seen to be most effective against methicillin-resistant *S. aureus* (MRSA) in these assays, though it did not reach statistical significance. Also, neither *K. pneumoniae* nor either of two *A. baumanii* strains were affected in the presence of the three lead compounds tested, though compound **5** did present a trend towards reduced biofilm in the #109 strain. The inter-species differences observed with respect to *Staphylococcus* led us to investigate the activity of these compounds further against a panel of environmental and clinical isolates to determine how phenotypic heterogeneity would present in the case of growth antagonism and/or anti-biofilm activity.

### Strain- and species-specific control of biofilm and growth in clinical *Staphylococci* spp*.*

**Fig. 6.**
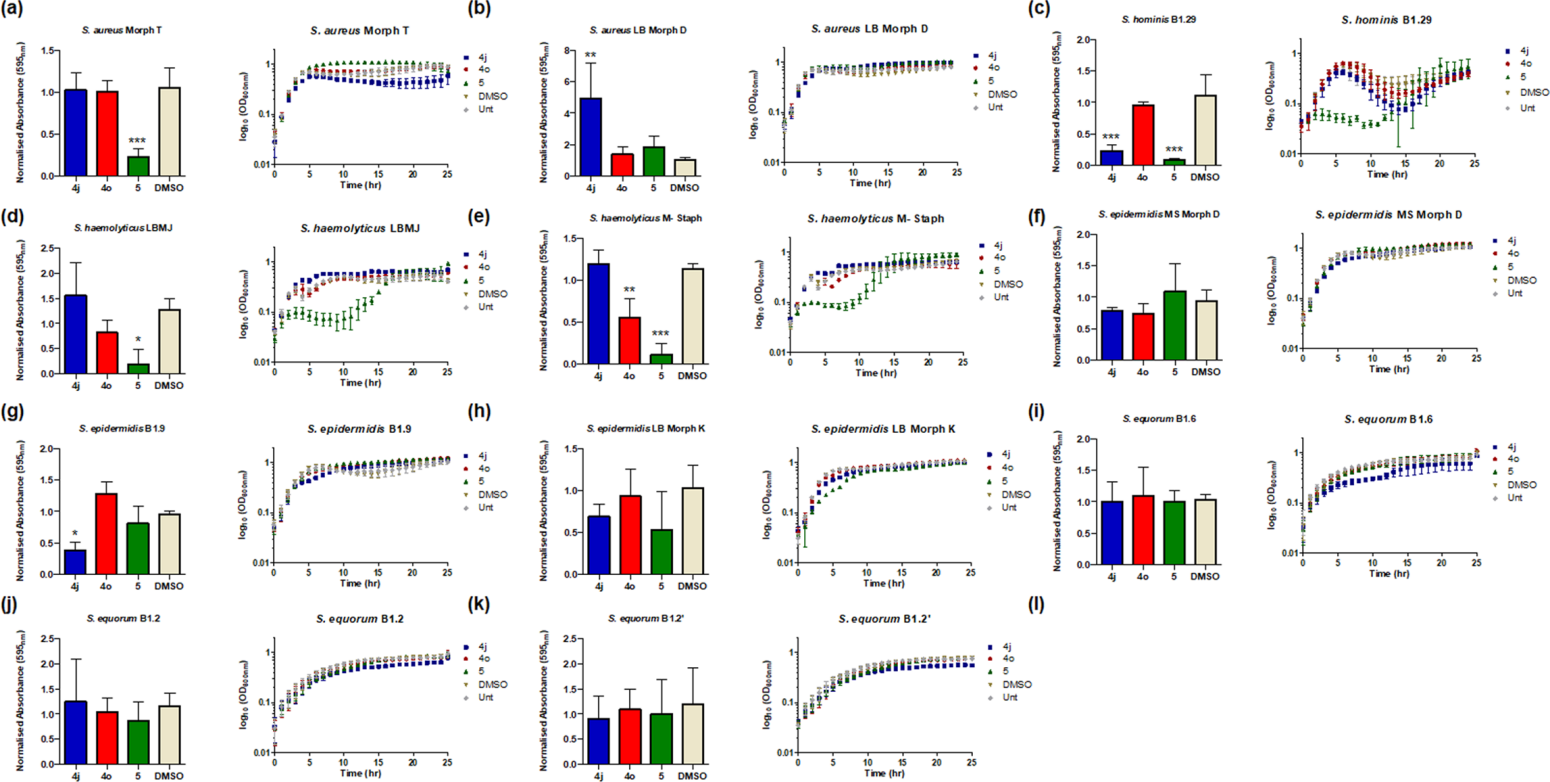
Strain-level divergence of compound efficacy against (i) biofilm formation and (ii) growth kinetics of *Staphylocccus* species, with model and clinical isolates: (**a-c**) *S. aureus *, (**d–f**) *S. haemolyticus *, (**g–i**) *S. epidermidis *, (**j–l**) *S. equorum* and (**m**) *S. hominis *. All data presented are the mean (±sem) of at least three independent biological replicates performed on 24-well plates. Statistical analysis was performed by one-way ANOVA with Dunnett’s Multiple Comparison test (*P≤0.05, **P≤0.005, ***P≤0.001).

To explore the spectrum of activity of these compounds, and to determine whether or not the observed activity reflects species- or even strain-specific interactions, we tested the lead compounds **4j**, **4o** and **5** against a collection of *Staphylococcus* species, including *S. hominis* and *S. equorum *, and a panel of clinical *S. aureus *, *S. haemolyticus* and *Staphylococcus* spp. (Fig. 6). A significant degree of inter-strain and inter-species phenotypic heterogeneity was observed with respect to the anti-biofilm and anti-growth activity of these compounds. While *S. equorum* biofilm appeared unaffected in the presence of any of the three compounds, strains of *S. haemolyticus *, *S. epidermidis* and *S. aureus* displayed varying degrees of susceptibility. Compound **5** was antagonistic to growth of all three *S. epidermidis* strains tested, along with *S. hominis *, and the apparent reduction in biofilm formation in these strains probably reflects this. On the other hand, compound **4j**, which was antagonistic to *B. atropheaus* biofilm formation, also suppressed biofilm formation in *S. hominis* and in one of the clinical *S. epidermidis* strains.

### Swarming inhibition in *P. aeruginosa* and *B. atropheaus* by lead HHQ derivatives

Another multicellular behaviour closely associated with the early stages of the host–pathogen interaction, but often ‘lost’ following colonization, is swarming motility. Distinguished from other forms of motility on the basis that it requires cell–cell communication, swarming motility is an important marker for virulence behaviour in pathogens. Previously, HHQ has been shown to be required for swarming motility in *P. aeruginosa* and it has been shown to suppress swarming motility in *B. atropheaus*. Therefore, we investigated whether our suite of compounds could interfere with swarming motility in these species.

**Fig. 7.**
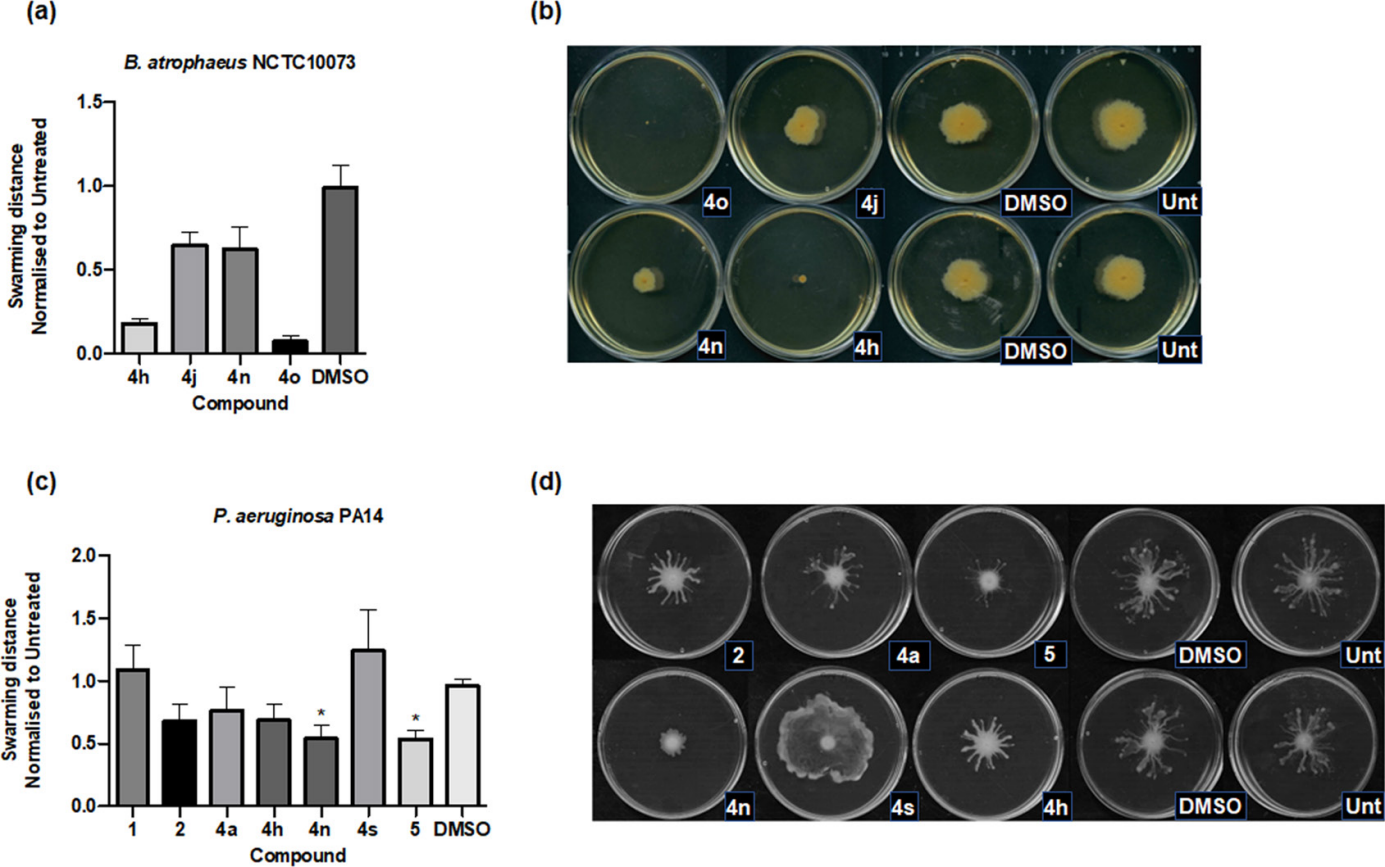
Swarming interference by derivative compounds. (**a**) Swarm distance in *B. atropheaus* (*n*=2 independent biological replicates); (**b**) visual representation of *B. atropheaus* swarming motility; (**c**) swarm distance in *P. aeruginosa* PA14 (*n*=3 independent biological replicates); (**d**) visual representation of *P. aeruginosa* swarming motility. Data are normalized relative to the untreated control (±sem). Significant differences were determined by one-way ANOVA with Dunnett’s Multiple Comparison test. Asterisks represent statistically significant differences relative to the untreated control (*P≤0.05).

An initial screen of the 23 compounds against *B. atrophaeus* NCTC 10073 and *P. aeruginosa* PA14 identified several compounds that were potent enough to suppress swarming motility in one or both of the species tested. In the case of *B. atrophaeus,* compounds **4h**, **4j**, **4n** and **4o** had significantly smaller swarm diameters than the DMSO control, with repression of swarming motility being most evident in plates treated with compounds **4o** and **4h** (Figs 7 and S3). However, it should be noted that compounds **4o** and **4j** were subsequently found to exhibit a negative influence on the growth of *B. atropheaus* (Fig. S4), suggesting that the repression of swarming may simply be a result of antibacterial activity. Visualization and measurement of the swarm diameter in *P. aeruginosa* PA14 revealed that only compounds **5** and **4n** were effective in suppressing this activity. Compound **4s** resulted in an unusual swarming behaviour, whereby swarming did not present as the typical tendril formation but rather was a film-like spread on the plate (Fig. 7). This suggests that compound **4s** may disrupt the direction and control of swarming behaviour but not the ability of *P. aeruginosa* to traverse the plate from the point of inoculum.

### Structural analysis of benzofuroquinolines as virulence inhibitors

The phenotypic patterns identified at the strain and species level in response to benzofuroquinoline challenge suggest a significant degree of phenotypic and genotypic heterogeneity amongst the ESKAPE pathogens tested. However, a number of derivative compounds were shown to possess cross-species activities, with compound **5** being most notable. Suppression of biofilm formation and swarming motility in *P. aeruginosa* was matched by growth inhibition and anti-biofilm activity against several *Staphylococcus* species, with anti-biofilm activity also observed against *E. faecalis *. Compound **5** carries a distinctive N substitution in the pyran ring, which may contribute to its broad-spectrum activity. Compounds **2**, **4a** and **4n** were effective at suppressing biofilm formation, swarming motility and pyocyanin production in *P. aeruginosa *, though none were particularly effective against other ESKAPE pathogens. These compounds contain either a methyl or H group at the alkyl chain position, with the latter two compounds also carrying an MeO substitution on opposing phenoxy and quinoline rings. Another MeO-substituted compound (**4o**), differing from **4n** only by the position of the substitution on the quinoline, exhibited significant growth-independent anti-biofilm activity against *S. haemolyticu* s, and suppressed swarming motility in *B. atropheaus* with growth antagonism evident in this species. Interestingly, compound **4n** suppressed both biofilm formation and swarming motility in *B. atropheaus*, suggesting the position of the MeO substitution may have a key role in this interaction. The finding that a significant number of compounds had no effect on the test strains under assay conditions would suggest that there is a strong requirement for structural fidelity in the framework used. Halogenated substitutions in particular were least effective, with compounds carrying F or Cl substitutions exhibiting little or no activity against test pathogens under the assay conditions used in this study.

## Discussion

Microbial infections, especially chronic infections such as the co-infection of *S. aureus* and *P. aeruginosa* in cystic fibrosis, represent a significant burden on health systems and are often recalcitrant to antibiotic treatment [44]. While the intricate molecular mechanisms that govern temporal relationships between species within these mixed communities present a significant unmet knowledge gap, they also present a unique opportunity for control of microbial behaviours through chemical communication [45]. Quinolone-based biofilm inhibitors in *P. aeruginosa* have previously been described, with phthalazine – quinolines [46] and aminoquinolines [47] exhibiting good efficacy. Previously, compound **1** had been shown to be unable to activate PqsR signalling, as seen through *pqsA* promoter fusion analysis and pyocyanin restoration assays [42]. However, in this study we show that compound **1** can antagonize biofilm formation in *P. aeruginosa *, as does the quinoline with a shorter methyl chain [2]. Loss of the alkyl chain resulted in a complete loss of anti-biofilm activity, but this was surprisingly recovered by methoxy substitution of the phenoxy ring (**4a**). On the other hand, both compounds **2** and **3** were able to suppress swarming motility in *P. aeruginosa *, while the full-length alkyl chain compound **1** did not. Espinosa-Valdés *et al*. have previously shown that shortening the alkyl chain of 2-amino-4-quinolone (AQ) derivatives can lead to loss of activity against *P. aeruginosa* biofilms [48]. Several studies have shown that a wide range of structural modifications in AQ analogues are capable of enhancing or maintaining anti-biofilm, anti-swarming and anti-pyocyanin production activities [30, 48–52], suggesting promiscuity within the framework from an antagonistic perspective.

The AQ class of signal molecule has also previously been shown to be an effective modulator of non-self behaviours, in both bacteria and fungi [23, 27–30]. Controlling key virulence phenotypes such as biofilm formation, motility, pigmentation, and antibiotic tolerance in pathogens with which *P. aeruginosa* is known to co-colonize, this class of molecular signal has emerged as a key inter-species and inter-kingdom communication system. It is encouraging therefore that the anti-virulence activity of the compounds described in this study also extends to Gram-negative and Gram-positive pathogens outside of the genus *Pseudomonas *. In this study we show that compounds with no long alkyl chain (**2** and **5**) and those substituted with an electron-donating OCH_3_ group [53] (**4n** and **4o**) are active against virulence-associated behaviours in ESKAPE pathogens. It is interesting that substitution at the same position with an electron-withdrawing CF_3_ group [54] (compare **4n** and **4s**) leads to differing activity, and thus both location and electronics play a role. Previous reports have described potent agents against staphylococcal nosocomial infectious bacteria, such as small quinoline molecules against *S. aureus* dispersion and *S. epidermidis* biofilm formation [55], and the action of halogenated quinolines against *S. epidermidis* [56]. Compounds of the indoloquinoline class have previously been shown to possess antibacterial activity when tested against MRSA [57]. Most closely resembling compound **5**, this suggests there is significant scope for development and modification of the indoloquinoline framework towards control of microbial infection. Khamkhenshorngphanuch *et al*. have also reported promising MIC data for 4-hydroxy-2-quinolinone analogues against *S. aureus *, *Escherichia coli* and *Aspergillus flavus* [58]. Structure–activity analysis revealed that the type of substituent has an impact on antimicrobial activities as does the length of the alkyl chain [58], adding to the growing understanding of AQ functionality with respect to cell–cell signalling and behavioural control of competing pathogens [30, 49–51, 59, 60].

The relatively few existing studies on the interspecies activity of the AQ signalling framework and its ability to control the virulence profiles of other microbes means that there is currently no concise understanding with respect to the possible ligand–receptor interactions that underpin AQ-mediated interspecies communication. While compounds with a shorter alkyl chain have been shown to bind more effectively to the LasR quorum sensing receptor protein than even the agonist signal molecule [48], there is no evidence in this current study of receptor binding at the interspecies level [44, 61]. Co-infection between *P. aeruginosa* and *S. aureus* promotes changes in genetic regulation resulting in a conversion of essential genes to expendable genes in co-infection, as observed in a murine wound model [44]. Furthermore, a proto-cooperative and enhancing relationship between these two important nosocomial pathogens have also been reported, highlighting the complex relationships that occur spatially and temporally during infection and chronic colonization [62, 63]. It has also recently been shown that PQS interacts with non-PqsR proteins in the cell, suggesting that virulence modulation may extend to other indirectly related systems or even unknown systems [64, 65]. Therefore, elucidating the molecular basis for the strain-specific antagonism identified in this study will be complex. Plotting evolutionary distances and mapping genetic drift to increasing or indeed decreasing compound susceptibility will be important if tailored application of compounds at the strain level is to be achieved or even desired. Furthermore, studies using multiple models of infection will be required to investigate the molecular basis of these interactions and to understand the dynamics of the underlying microbial interactions during chronic human infection [44, 66]. Developing and optimizing these infection models remains challenging due to their complexity, and a strong multidisciplinary effort will be required.

The ultimate application for anti-virulence approaches such as that described here may be to act in concert with existing antimicrobial interventions. This is particularly the case where the action of these antibiotic agents is inhibited or lessened by virtue of the biofilm lifestyle of pathogenic organisms [67]. Notwithstanding the potential for this combinatorial approach, examples of successes in this realm beyond *in vitro* studies are limited. Further work is required to understand how anti-virulence approaches operate within complex microbiomes, in a context where strain diversity may match, or even surpass, species diversity [68]. It should also be noted that the switch between a biofilm and free-living planktonic lifestyle is tightly co-ordinated [69]. It follows that while biofilm prevention may be key to unlocking the potential of existing and new antimicrobial chemical entities, promoting acute planktonic virulence behaviour may be an unintended consequence of this. This is an important consideration that needs to be addressed in the design of anti-virulence strategies.

## Supplementary Data

Supplementary material 1
