# Supplementary material for: Structural modification of the Pseudomonas aeruginosa alkylquinoline cell–cell communication signal, HHQ, leads to benzofuranoquinolines with anti-virulence behaviour in ESKAPE pathogens
Source: Microbiology (Reading). 2023 Mar 2;169(3):001303. doi: 10.1099/mic.0.001303 (PMC10191377; doi:10.1099/mic.0.001303)
Supplement: Supplementary material 1 [file mic-169-1303-s001.pdf]

Research Article: Microbiology

Structural modification of the *Pseudomonas aeruginosa* alkylquinoline cell-cell communication signal, HHQ, leads to benzofuranoquinolines with anti-virulence behaviour in ESKAPE pathogens

Veronica Rossetto[1,2], Ay'sha Moore-Machacek[2], David F. Woods[2], Helena M. Galvão[1], Rachel M. Shanahan[4], Aobha Hickey[4], Niall O'Leary[2], Fergal O'Gara[2,3,5], Gerard P. McGlacken[4,6], and F. Jerry Reen[2,6*].

Supplementary Data Figures

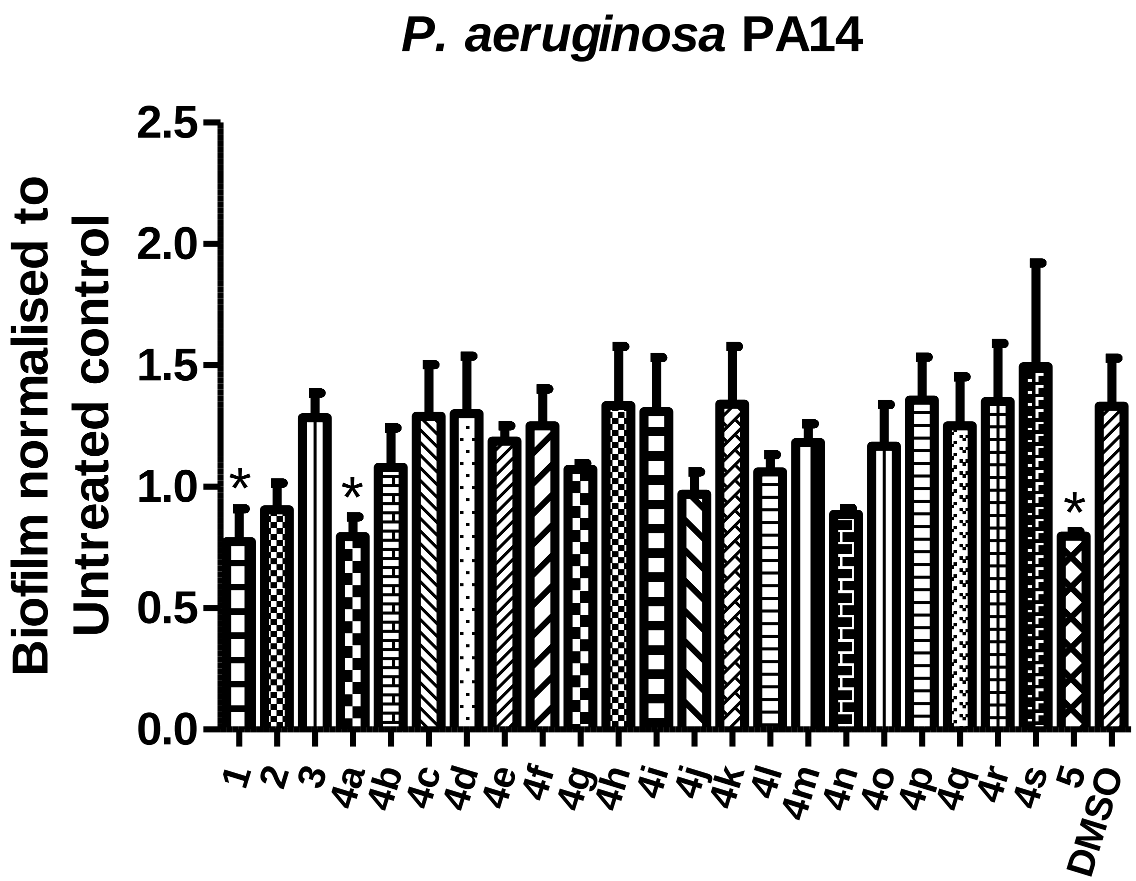

Supplementary Figure S1. Biofilm formation in *P. aeruginosa* PA14 in the presence of benzofuroquinoline compounds. All data presented is the mean (+/- SEM) of at least 3 independent biological replicates. Statistical analysis was performed by Student's paired t-test ($*p \leq 0.05$, $** p \leq 0.005$).

(a)

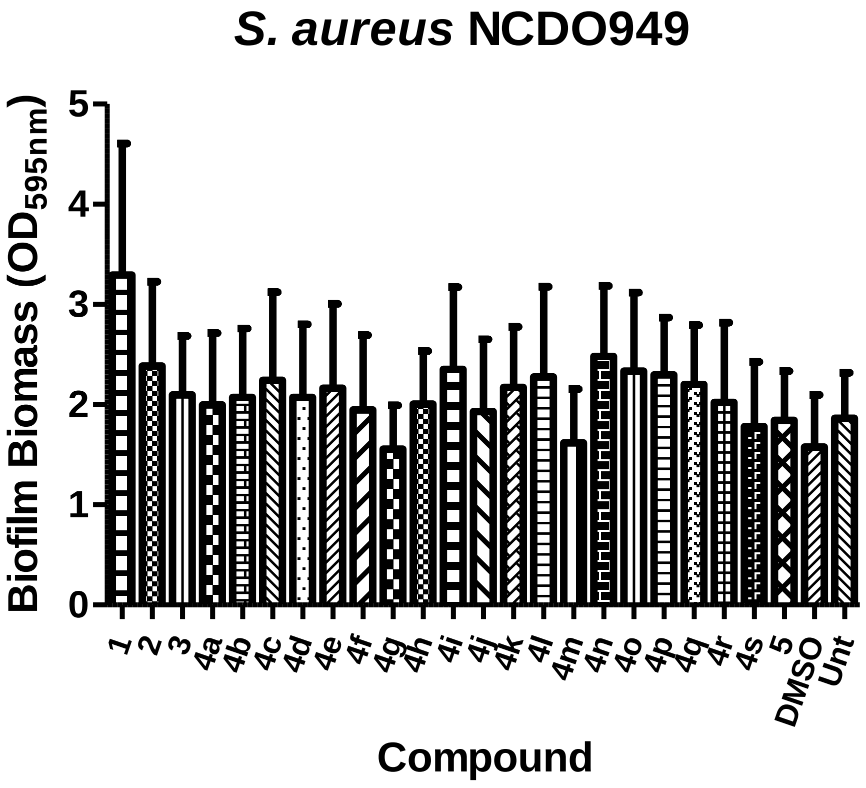
S. aureus NCDO949
Biofilm Biomass (OD_{595nm})
0
1
2
3
4
5
1 2 3 4a 4b 4c 4d 4e 4f 4g 4h 4i 4j 4k 4l 4m 4n 4o 4p 4q 4r 4s 5 DMSO Unt
Compound

(b)

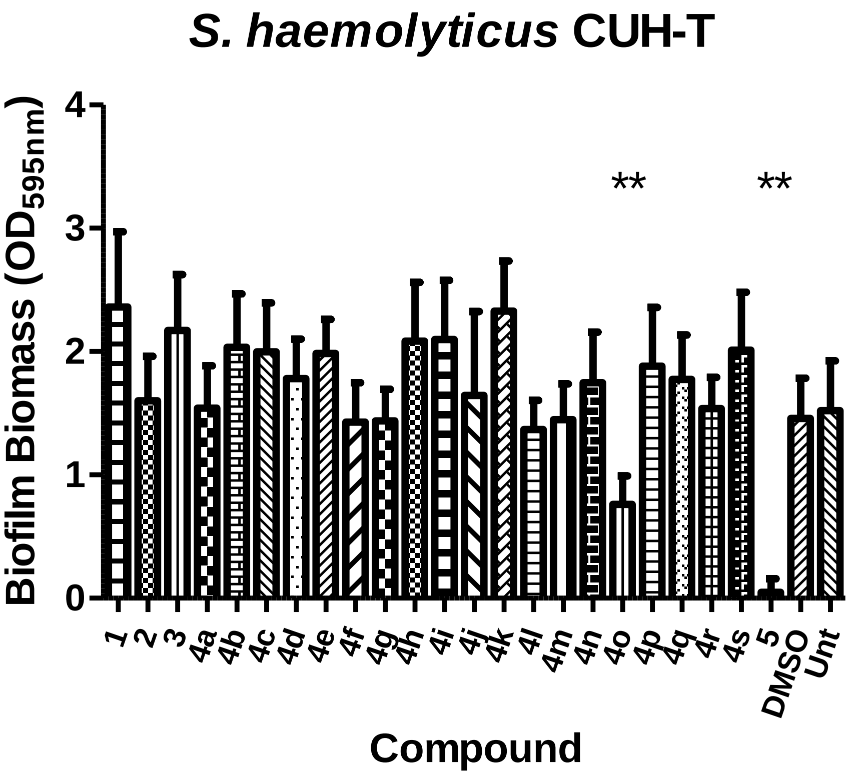
S. haemolyticus CUH-T
Biofilm Biomass (OD_{595nm})
0
1
2
3
4
**
**
1 2 3 4a 4b 4c 4d 4e 4f 4g 4h 4i 4j 4k 4l 4m 4n 4o 4p 4q 4r 4s 5 DMSO Unt
Compound

(c)

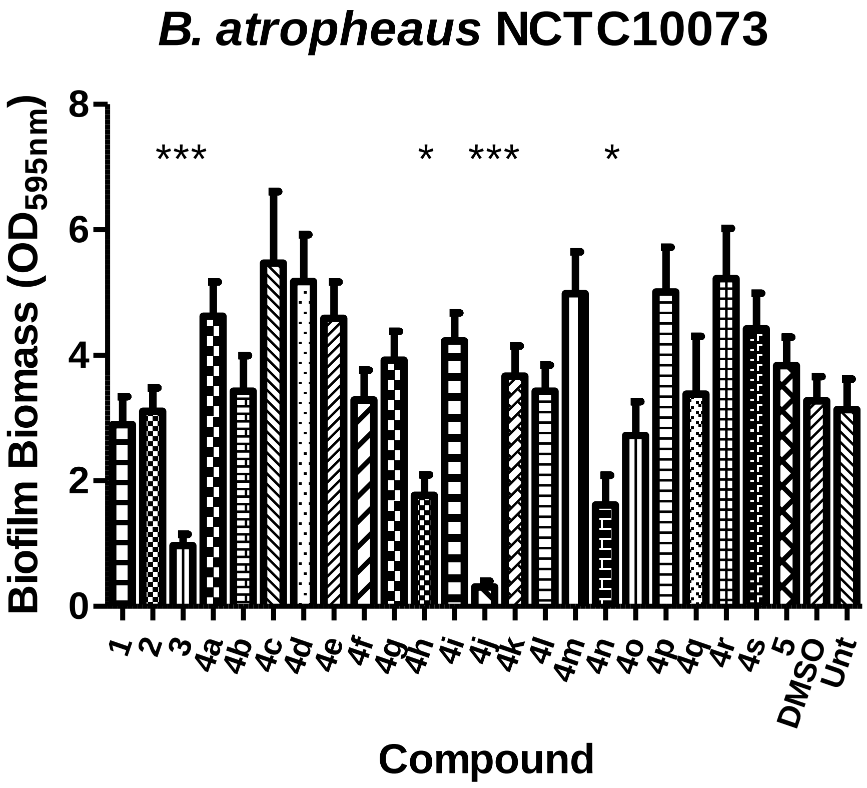

Supplementary Figure S2. Biofilm formation in (a) *S. aureus* NCDO 949, (b) *S. haemolyticus* CUH-T, and (c) *B. atropheaus* NCTC10073 in the presence of benzofuroquinoline compounds. All data presented is the mean (+/- SEM) of at least 3 independent biological replicates. Statistical analysis was performed by Student's paired t-test (*$p \leq 0.05$, ** $p \leq 0.005$, *** $p \leq 0.001$).

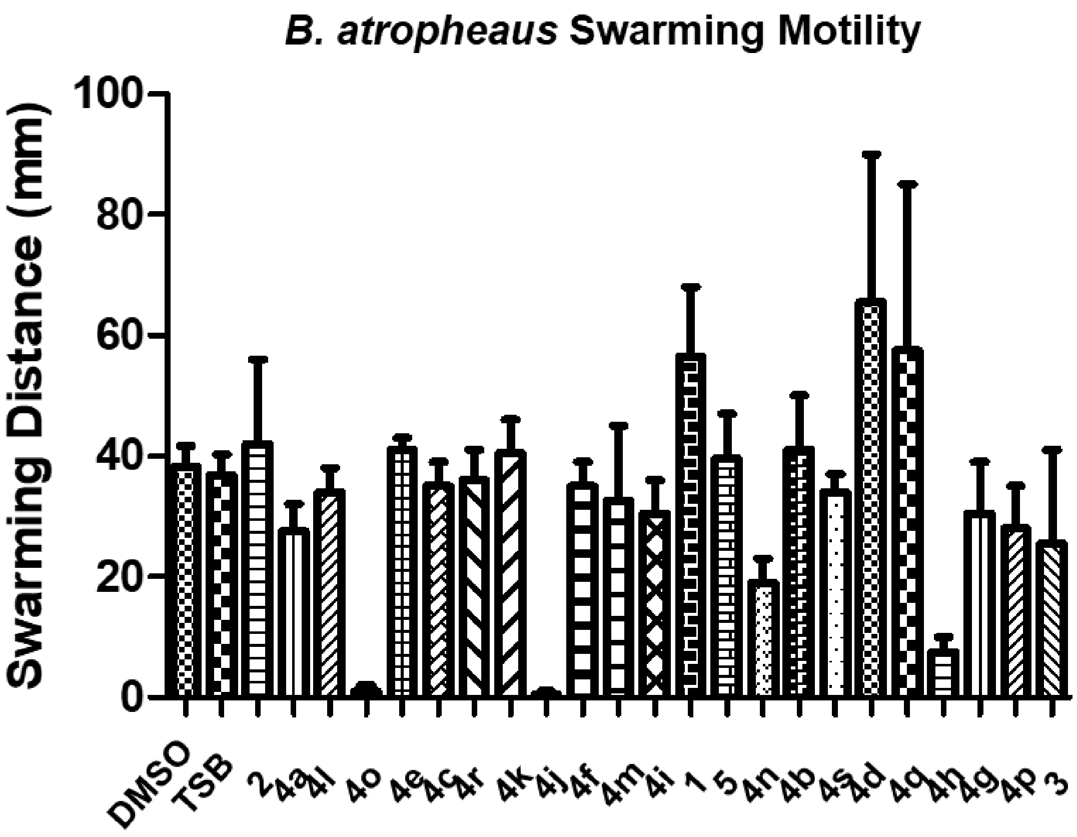

Supplementary Figure S3. Swarming motility of *B. atropheaus* NCTC10073 in the presence of benzofuroquinoline compounds. All data presented is the mean (+/- SEM) of 2 independent biological replicates.

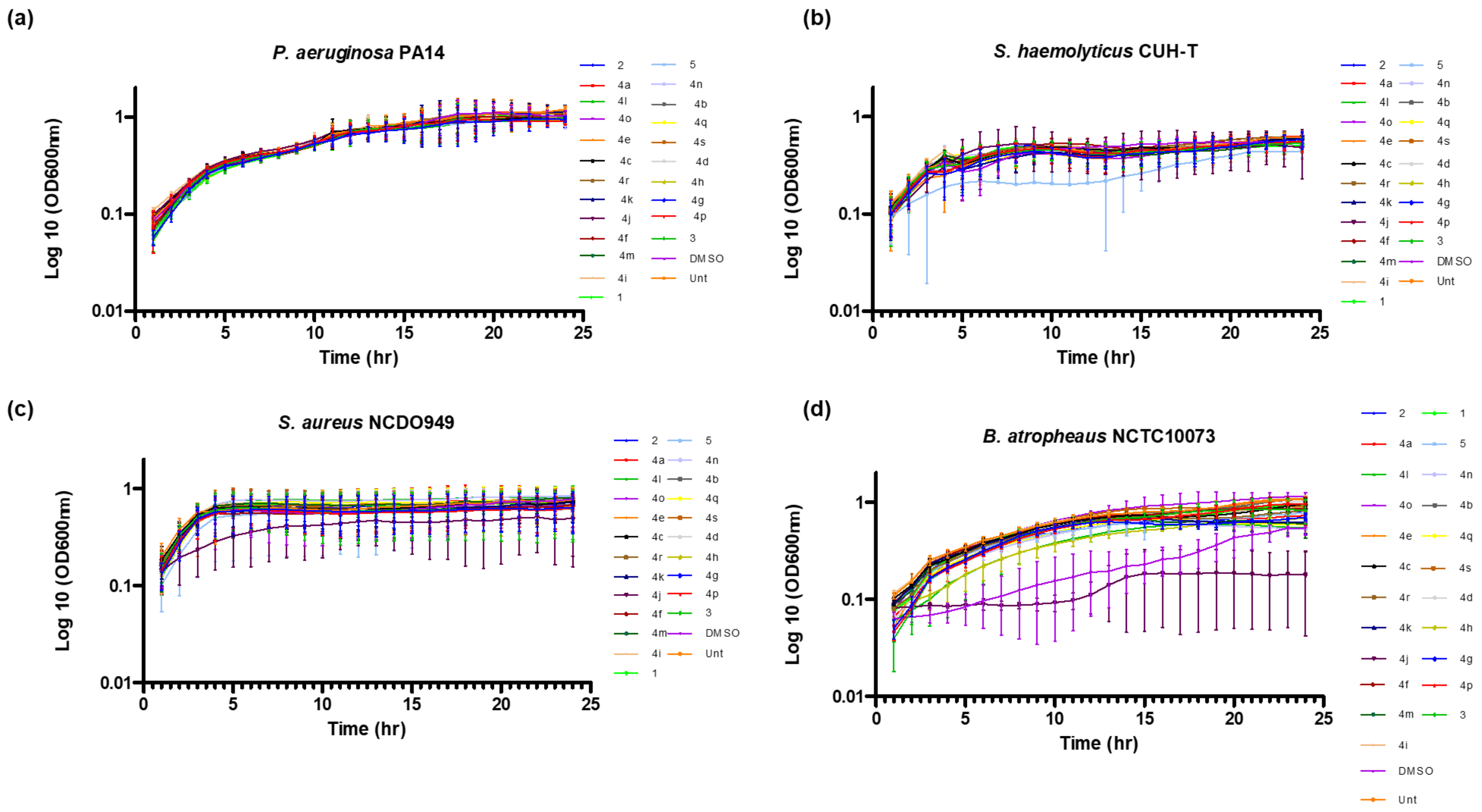

Supplementary Figure S4. Kinetic growth curve analysis of (a) *P. aeruginosa* PA14, (b) *S. haemolyticus* CUH-T, (c) *S. aureus* NCDO 949, and (d) *B. atropheaus* NCTC10073 in the presence of benzofuroquinoline compounds. All data presented is the mean (+/- SEM) of 3 independent biological replicates.

Individual Data point entries for Figures 2-7. Graphs correspond to data represented as mean (+/-SEM) bar graphs in the manuscript and is provided here for completeness.

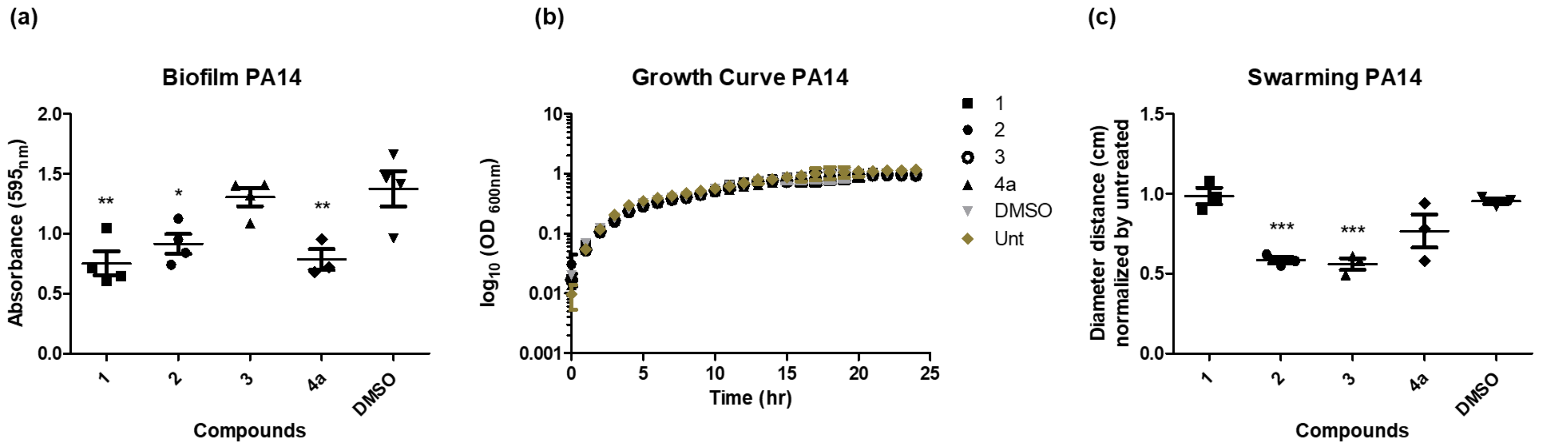

Figure 2. Activity analysis of benzofuroquinoline compounds (30 µM) against *P. aeruginosa* PA14. (a) Biofilm assay presented as crystal violet biomass Abs595nm; (b) Growth curve analysis in the presence of compounds and DMSO carrier control; (c) Swarming motility on Eiken Agar plates measured as the swarm distance from the tips of the outermost tendrils characteristic of this strain and normalised to the untreated control. In all cases, data represents the mean (+/-SEM) of at least three independent biological replicates. Statistical significance (one-way ANOVA with Dunnetts Multiple Comparison test) is presented relative to the DMSO carrier control ($*p \leq 0.05$, $**p \leq 0.005$, $***p \leq 0.001$).

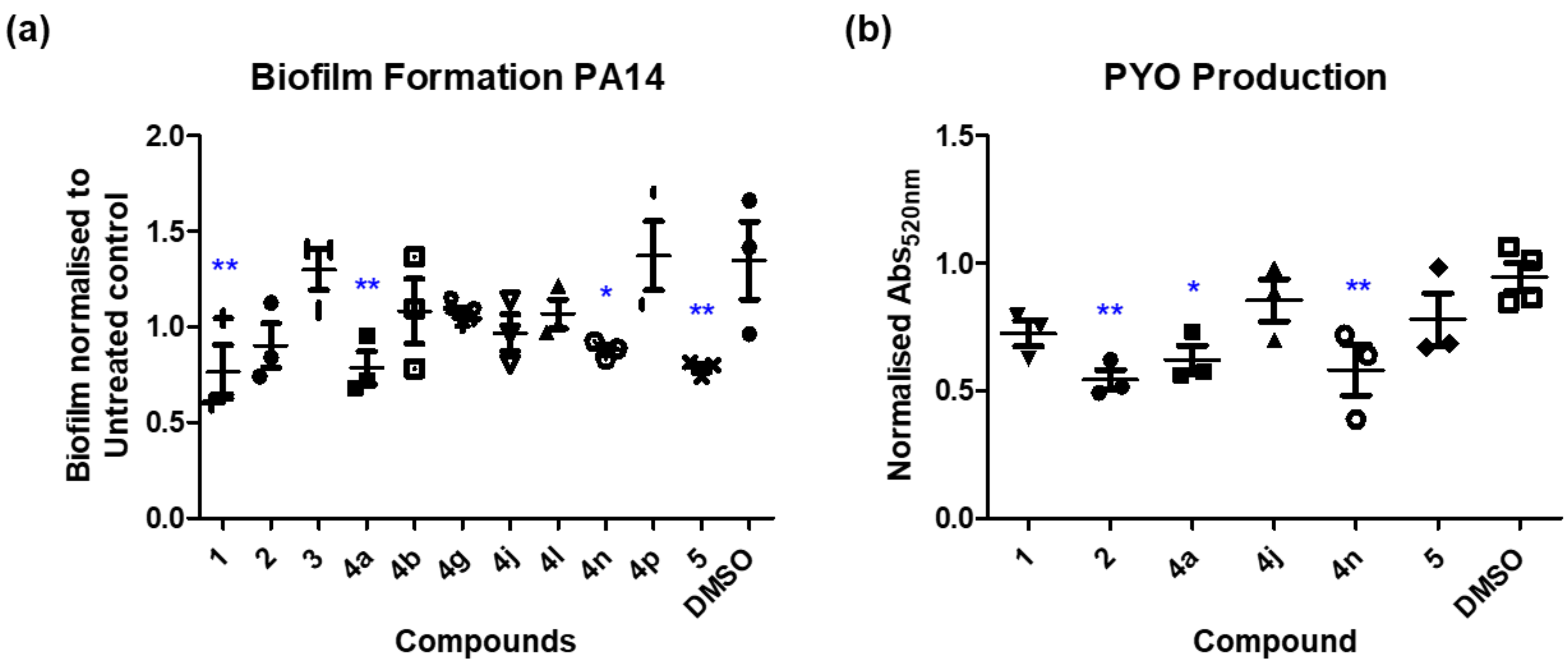

Figure 3. (a) Biofilm assay normalised to untreated control; (b) pyocyanin extraction normalised to the untreated control. Data presented is the mean (+/-SEM) of at least three independent biological replicates. Significant differences were determined by one-way ANOVA with Dunnett's multiple comparison test ($*p \leq 0.05$, $**p \leq 0.005$).

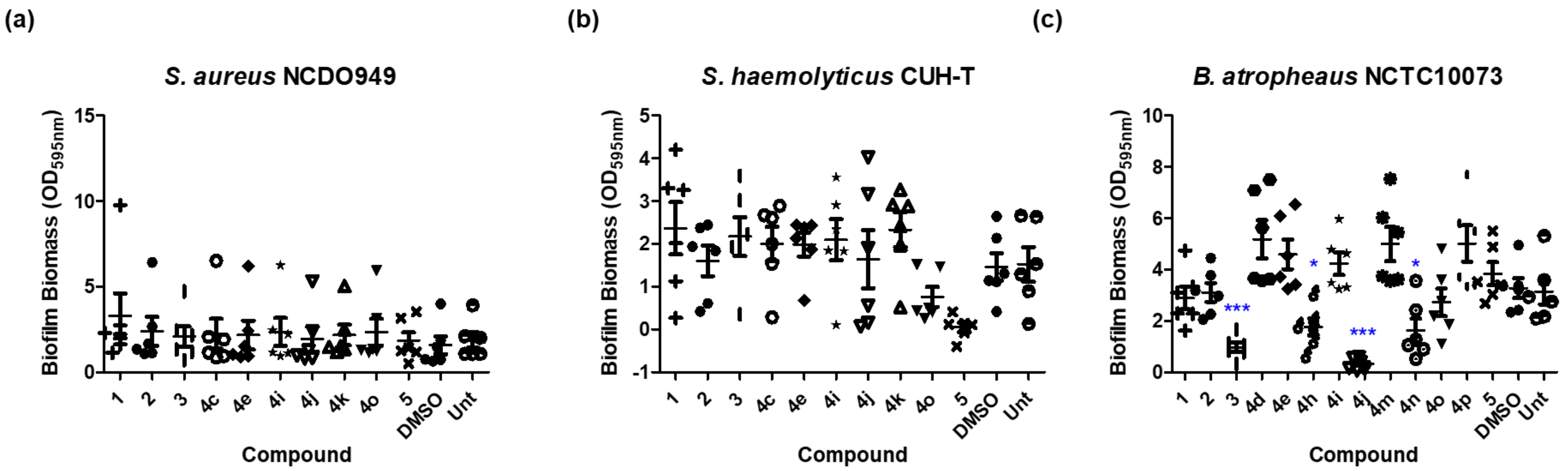

Figure 4. Biofilm formation in (a) *S. aureus*, (b) *S. haemolyticus* and (c) *B. atropheaus* strains as measured in 96-well plates with crystal violet staining. All data presented is the mean (+/- SEM) of at least 5 independent biological replicates. Statistical analysis was performed by one-way ANOVA with Dunnetts Multiple Comparison test (* $p \leq 0.05$, *** $p \leq 0.001$). In each panel, Unt refers to the untreated control.

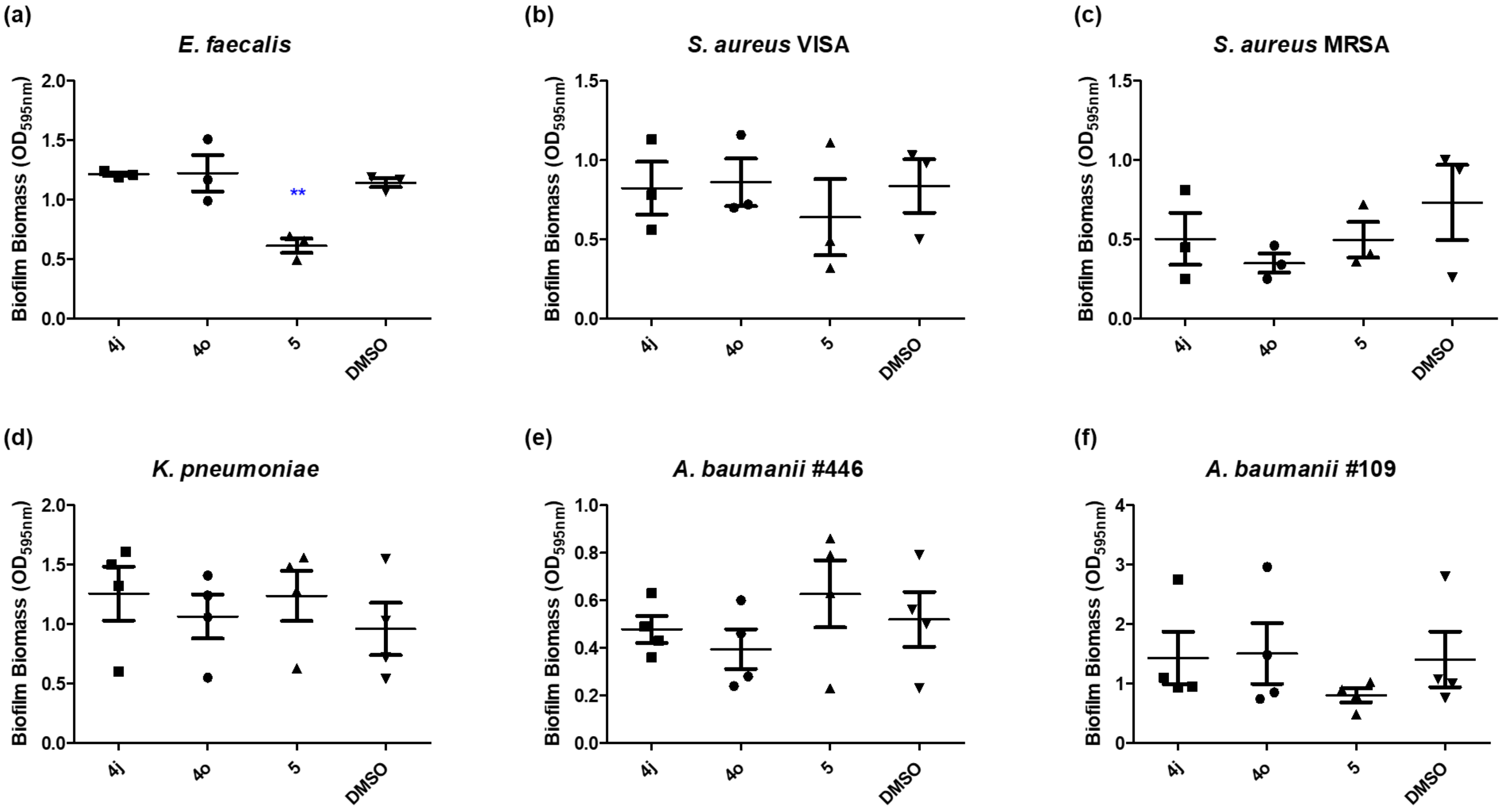

Figure 5. Biofilm formation in *E. faecalis* and ESKAPE pathogens in the presence of lead compounds (30 µM) 4j, 4o, and 5. All data presented is the mean (+/- SEM) of at least 3 independent biological replicates. Statistical analysis was performed by one-way ANOVA with Dunnett's Multiple Comparison testing (** $p \leq 0.005$).

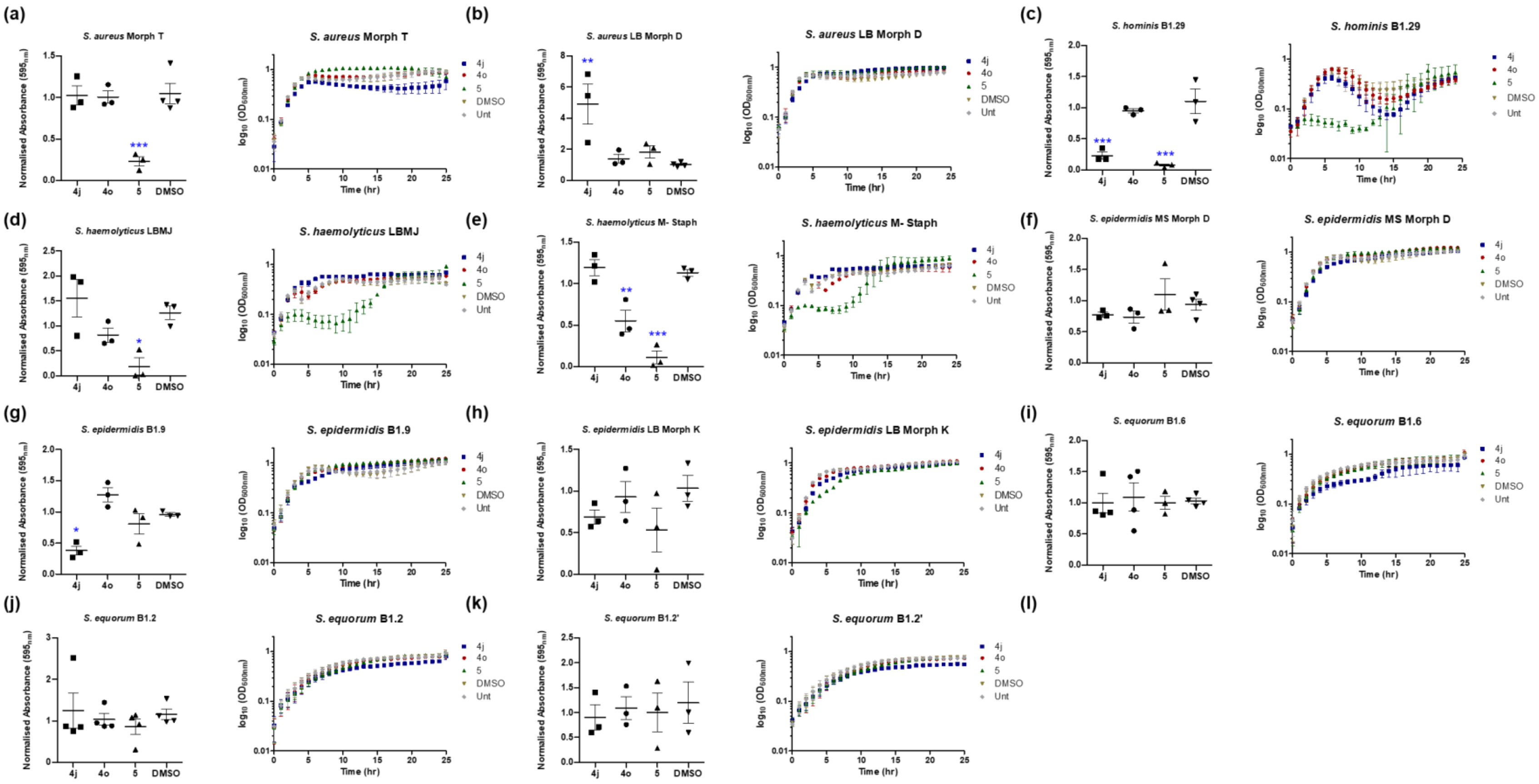

Figure 6. Strain-level divergence of compound efficacy against (i) biofilm formation and (ii) growth kinetics of Staphylocccus species, with model and clinical isolates: (a-c) *S. aureus*, (d-f) *S. haemolyticus*, (g-i) *S. epidermidis*, (j-l) *S. equorum* and (m) *S. hominis*. All data presented is the mean (+/- SEM) of at least three independent biological replicates performed on 24 well plates. Statistical analysis was performed by One-Way ANOVA with Dunnett’s Multiple Comparison testing.

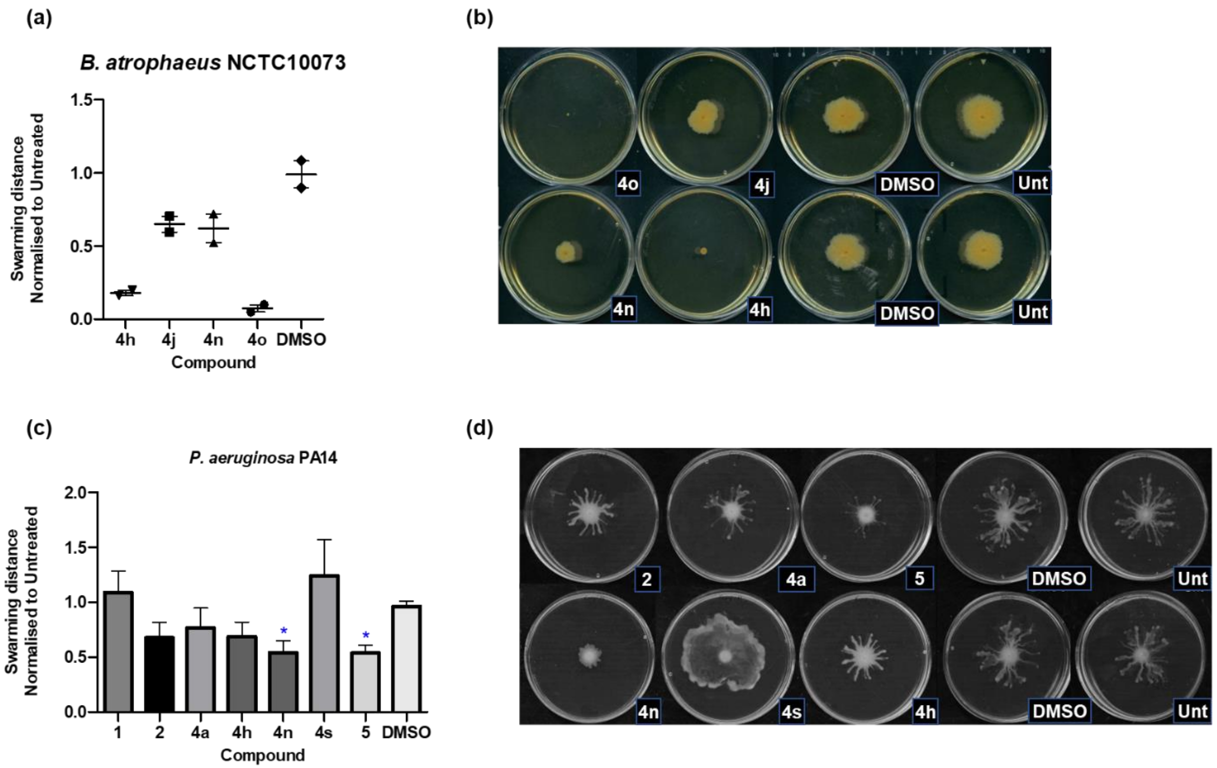

Figure 7. Swarming interference by derivative compounds. (a) Swarm distance *B. atropheaus* (n=2 independent biological replicates; (b) Visual representation of *B. atropheaus* swarming motility; (c) Swarm distance *P. aeruginosa* PA14 (n=3 independent biological replicates); (d) visual representation of *P. aeruginosa* swarming motility. Data is normalised relative to the untreated control (+/- SEM). Significant differences were determined by one-way ANOVA with Dunnett's Multiple Comparison test. Asterisks represent statistically significant differences relative to the untreated control ($*p \leq 0.05$).
